# Modeling analysis of armed conflict risk in sub-Saharan Africa, 2000–2019

**DOI:** 10.1371/journal.pone.0286404

**Published:** 2023-10-02

**Authors:** Xiaolan Xie, Dong Jiang, Mengmeng Hao, Fangyu Ding

**Affiliations:** 1 State Key Laboratory of Resources and Environmental Information System, Institute of Geographic Sciences and Natural Resources Research, Chinese Academy of Sciences, Beijing, China; 2 College of Resources and Environment, University of Chinese Academy of Sciences, Beijing, China; 3 Key Laboratory of Carrying Capacity Assessment for Resource and Environment, Ministry of Land & Resources, Beijing, China; Utah State University, UNITED STATES

## Abstract

Sub-Saharan Africa has suffered frequent outbreaks of armed conflict since the end of the Cold War. Although several efforts have been made to understand the underlying causes of armed conflict and establish an early warning mechanism, there is still a lack of a comprehensive assessment approach to model the incidence risk of armed conflict well. Based on a large database of armed conflict events and related spatial datasets covering the period 2000–2019, this study uses a boosted regression tree (BRT) approach to model the spatiotemporal distribution of armed conflict risk in sub-Saharan Africa. Evaluation of accuracy indicates that the simulated models obtain high performance with an area under the receiver operator characteristic curve (ROC-AUC) mean value of 0.937 and an area under the precision recall curves (PR-AUC) mean value of 0.891. The result of the relative contribution indicates that the background context factors (i.e., social welfare and the political system) are the main driving factors of armed conflict risk, with a mean relative contribution of 92.599%. By comparison, the climate change-related variables have relatively little effect on armed conflict risk, accounting for only 7.401% of the total. These results provide novel insight into modelling the incidence risk of armed conflict, which may help implement interventions to prevent and minimize the harm of armed conflict.

## 1. Introduction

As one of the greatest threats to humans, armed conflict has endangered lives and property and caused considerable damage across the world, especially in sub-Saharan Africa [1, 2]. According to the Armed Conflict Location and Event Dataset (ACLED) record, the number of battle events in sub-Saharan Africa has been increasing from 1709 in 2000 to 3969 in 2019, with tens of thousands of people injured and killed annually [3]. In addition to causing immediate casualties, armed conflict might result in people losing economic opportunities and becoming destitute and homeless, further affecting millions more [4]. Given the magnitude of the damage caused by armed conflict, understanding the driving factors of armed conflict and modelling the incidence risk of armed conflict have been major projects for both academia and policy.

Armed conflict is a complex and multifaceted phenomenon whose incidence is driven by a wide range of factors [5]. Early studies on the drivers of armed conflict focus on factors that represent politics, socioeconomic, and environmental [6–8]. For example, Hegre and Sambanis [9] systematically explored the impact of multiple variables on armed conflict and found that countries with a large population and low income levels had a higher level of armed conflict. Based on geographical information systems (GIS) technology, Buhaug and Lujala [10] explored the relationship between seven geographical variables, including mountains and forests, and armed conflict. By examining various aspects and outstanding features of political liberalization and armed conflict, Takeuchi [11] stressed the important role of political liberalization in the incidence of armed conflict.

As sudden changes in temperature and precipitation are expected to become more frequent in some areas due to climate change, some researchers recognized climate change as a threat multiplier and linked it to armed conflict [12–14]. For example, using a panel regression that links climate change and conflict events, Burke et al. [15] found a strong historical positive correlation between local temperatures and armed conflict in sub-Saharan Africa. Hsiang et al. [4] assembled and analysed 60 studies on climate change and human conflict, revealing that one standard deviation from normal precipitation and temperature is associated with a 14% increase in conflict risk on average. A collective of prominent climate-conflict researchers from different disciplines assessed the link between climate change and armed conflict; they deemed that 3–20% of organized armed conflict risk has been influenced by climate over the last century [16].

Based on these influencing factors of armed conflict, some studies have tried to establish armed conflict simulation and prediction models at regional or national scales [17–19]. For example, regarding factors such as population size, infant mortality rates, and demographic composition as independent variables, Hegre et al. [20] developed a statistical model to forecast armed conflict towards 2050 at the national level, and their results suggested that the proportion of countries in the world engaged in internal armed conflict would continue to decline. According to indicators that have been quantified in the shared socioeconomic pathway (SSP) and representative concentration pathway (RCP) projections, Hoch et al. [21] used machine learning methods to project subnational armed conflict risk over Africa. They indicated a more peaceful future compared to the current conditions for SSP1-RCP2.6, while armed conflict is increased in most countries under the SSP3-RCP6.0 scenario.

Although previous studies have explored multiple drivers of armed conflict, there is still a lack of knowledge about their relative importance [22]. Moreover, modelling armed conflict risk at regional or national scales might ignore much important information [10]. Therefore, it is necessary to establish a simulation model of armed conflict at a finer scale by comprehensively considering armed conflict driving actors. In this study, we assemble a large database of armed conflict, climate change, politics, socioeconomic, and environmental factors covering the period 2000–2019 at a spatial resolution of 0.5° (approximately 55km). A boosted regression tree (BRT) modelling procedure was used to analyse the relative contribution of each armed conflict driver and simulate the spatiotemporal distribution of armed conflict risk in sub-Saharan Africa.

## 2. Materials and methods

### 2.1. Data

This paper included the dataset of armed conflict, climate change, and background context factors, which are shown in Table 1.

**Table 1:** List of datasets.

Indicators	Variable	Source
Armed Conflict	Armed Conflict	ACLED (https://acleddata.com/)
Climate Change	Temperature Anomaly	the University of East Anglia Climate Research Unit (https://catalogue.ceda.ac.uk/)
	Precipitation Anomaly	
Background Context	Population density	Gridded Population of the World, Version 4 (https://sedac.ciesin.columbia.edu/data/set/gpw-v4-population-density-rev11)
	Liberal Democracy Index	Varieties of Democracy (https://www.v-dem.net/data/the-v-dem-dataset/)
	Urban accessibility	[23]
	Mean precipitation	the University of East Anglia Climate Research Unit (https://catalogue.ceda.ac.uk/)
	Mean temperature	
	Normalized difference vegetation index	Terra Moderate Resolution Imaging Spectroradiometer (MODIS) Vegetation Indices Monthly (MOD13C2) Version 6.1 (https://lpdaac.usgs.gov/products/mod13c2v061/)
	Nighttime lights	[24]
	Exclusion	EPR-ETH dataset (https://dataverse.harvard.edu/dataverse/epr)
	Land cover	European Space Agency (ESA) GlobCover (http://due.esrin.esa.int/page_globcover.php)

#### 2.1.1. Armed conflict

The armed conflict data are obtained from ACLED (https://acleddata.com/) [3]. This dataset records detailed information (e.g., the day, month, year, latitude, and longitude of an event that took place) of each event. The database provides multiple types of conflict (e.g., battles, riots, protests, and attacks on civilians). In this study, we regard the battle event as a representation of armed conflict, which was defined as “must be violent events involving at least two armed and organized actors”. We first select the battle events of each year according to the identifier (Year) in the ACLED. Then, using GIS software, we assign the battle events to a grid cell of 0.5° on an annual basis and statistically calculate the number of events in each grid cell. Finally, we construct a binary battle incidence indicator that takes a value of 1 if there was a battle event in the respective grid cell between 2000 and 2019 and 0 otherwise.

#### 2.1.2. Driving factors

- Climate Change;

Some studies have suggested that deteriorating climatic conditions, such as higher temperatures and lower precipitation levels, are associated with an increased risk of conflict [25]. To explore the impact of climate change on armed conflict, we obtain temperature and precipitation data from the Climate Research Unit at the University of East Anglia Climate Research Unit (https://catalogue.ceda.ac.uk/). The dataset provides information on the geographical distribution of the monthly surface temperature at a grid cell of 0.5°. Based on this dataset, we calculate the annual levels of precipitation and temperature for each grid. Then, long-term (1970–1999) mean precipitation and temperature distribution data were generated. To obtain annual precipitation and temperature anomaly data, we used the relative anomaly quantified as a standard score [z score] to estimate the deviations from normal (Eq 1). As an example, we use the temperature anomaly, which represents deviations from the multiannual average temperature, including positive and negative deviations with opposite numerical signs. Among them, 0 represents the multiannual average temperature (normal temperature), ±1 represents 1 standard deviation from the normal temperature, i.e., 1 represents 1 positive deviation from the normal temperature, and -1 represents 1 negative deviation from the normal temperature. Positive temperature deviation denotes the degree to which the temperature is higher than normal, and negative temperature deviation denotes the degree to which it is lower than normal.

$$T_{ij*}=\frac{T_{ij}‐\mu_{Ti}}{\delta_{Ti}}$$ (Eq 1)

Where *T*_ij*_ is the temperature anomaly of grid *i* in year *j*, *T_ij_* is the annual temperature of grid *i* in year *j*, *μ_Ti_* is the annual average temperature of grid *i* covering the period 1970–1999, and *δ_Ti_* is the standard deviation of the annual temperature of grid *i* covering the period 1970–1999.

- Background Context Factors;

While climate change is often found to be linked to armed conflict, it is only one of several major causes [26]. Other background context factors, such as population density, social welfare, and the political system of a country, are also important causes of armed conflict [27, 28]. Therefore, this study includes the following datasets to represent background context factors that affect armed conflict.

Previous studies suggested that armed conflict was more likely to occur in places with a large population density and facilities convenience [29]. Therefore, this study includes the population density from the Gridded Population of the World, Version 4 (GPWv4) of NASA Socioeconomic Data and Applications Center (https://sedac.ciesin.columbia.edu/data/set/gpw-v4-population-density-rev11). The GPWv4 estimated human population density (number of persons per square kilometre) based on counts consistent with national censuses and population registers. Moreover, we include the travel time to cities of more than 50,000 inhabitants estimated by Weiss et al. [23] to represent urban accessibility. This dataset integrated several roads, railways, rivers, and land cover types within a GIS model; thus, it has high quality and availability.

Previous studies have shown that social welfare is associated with armed conflict [6]. Due to the lack of available income and welfare data at the 0.5° grid/year, we refer to the conflict studies of von Uexkull et al. [30] and use nighttime light (NTL) to measure social welfare. In general, the brighter (the greater the value of NTL) the lights at night, the more developed the economy and the better the social welfare. The NTL data used in this study are estimated by Li et al. [24], who harmonized the intercalibrated NTL observations from Defence Meteorological Satellite Program (DMSP) data [31] and the simulated DMSP-like NTL observations from Visible Infrared Imaging Radiometer Suite (VIIRS) data [32]. The harmonized data show the brightness of NTL is highly correlated to gross domestic product and electric power consumption [24, 33].

Previous literature has also shown that armed conflicts tend to occur in areas dependent on agriculture [30, 34]. Vegetation index and land cover data are often used to measure agricultural yield production [14]. In this study, to capture the extent of agricultural dependence, we obtain the normalized difference vegetation index (NDVI) from the Terra Moderate Resolution Imaging Spectroradiometer (MODIS) Vegetation Indices Monthly (MOD13C2) Version 6.1 product [35] and land cover from the European Space Agency (ESA) GlobCover [36].

Some factors related to national politics are also important drivers of armed conflict, especially the political system and the vulnerability of the political dimension of a country [37, 38]. Therefore, we include the liberal democracy index variable from the Varieties of Democracy (V-Dem) dataset as a proxy for estimating liberal democracy [39]. V-dem provided a multidimensional and decentralized dataset that reflects the complexity of the concept of democracy as a governing system that goes beyond simple elections. In addition, we include a binary exclusion variable that determine whether the group is excluded from national political processes according to the EPR-ETH dataset [40]. The EPR dataset lists all politically relevant ethnic groups worldwide since 1945 and provides data on the group’s access to executive power for each year.

### 2.2. Method

To model on the same spatial scale, we unify all datasets to a spatial resolution of 0.5°. Moreover, we set the armed conflict events data as the dependent variable and other data mentioned in the Data section as independent variables. Based on these datasets, we use the BRT modelling approach to analyse the relative contribution of each independent variable and simulate the spatiotemporal distribution of armed conflict risk. In addition, we use random forest [18, 19, 41], which is widely used in conflict modeling, as the baseline model to measure the performance of the BRT model in conflict simulation.

The BRT model is a robust machine learning method that combines tree-based recursive partitioning with the concept of boosting, improving the predictive performance of a single-tree model [42]. Moreover, the BRT model could fit complex nonlinear relationships and automatically deal with interaction effects between predictors [43]. Detailed information about the BRT modelling approach can be found elsewhere [44–46]. Therefore, we assume that the BRT modelling approach that has been used in several fields is suitable for modelling armed conflict data with a complex structure [47–54].

We used the R version 4.2.1 64-bit statistical computing platform to build the BRT analysis approach. To avoid overfitting during the training process, we apply the tenfold cross-validation method to divide the dataset into training samples and test samples. To be specific, the dataset was partitioned into 10 subsets, one subset was used as the testing set and the rest was used for the training set. We deployed the BRT modeling framework using the ’dismo’ and ’gbm’ packages based on the R statistical programming environment. The parameters of these packages were constantly adjusted during the training process according to the performance of the model, we finally set a tree complexity at 4, an initial number of trees at 50, a learning rate of 0.01, and a step size of 10. Furthermore, we identified the optimum number of trees by setting the maximum number of trees at 10000 and minimizing model holdout deviation with step tree addition. In the process of BRT training, as trees are added, there is an initial steep decline in prediction error followed by a more gradual approach to the minimum (S1 Fig). Other parameters are retained at their default values. In addition, we adopted the area under the receiver operator characteristic curve (ROC-AUC) and the area under the precision recall curves (PR-AUC) as accuracy evaluation indices to assess the performance of the investigated model. Moreover, the relative contribution (RC) indicator is used to quantify the contribution of each independent variable to the spatiotemporal distribution of armed conflict risk.

## 3. Results

### 3.1. Armed conflict events statistics

**Fig 1:**
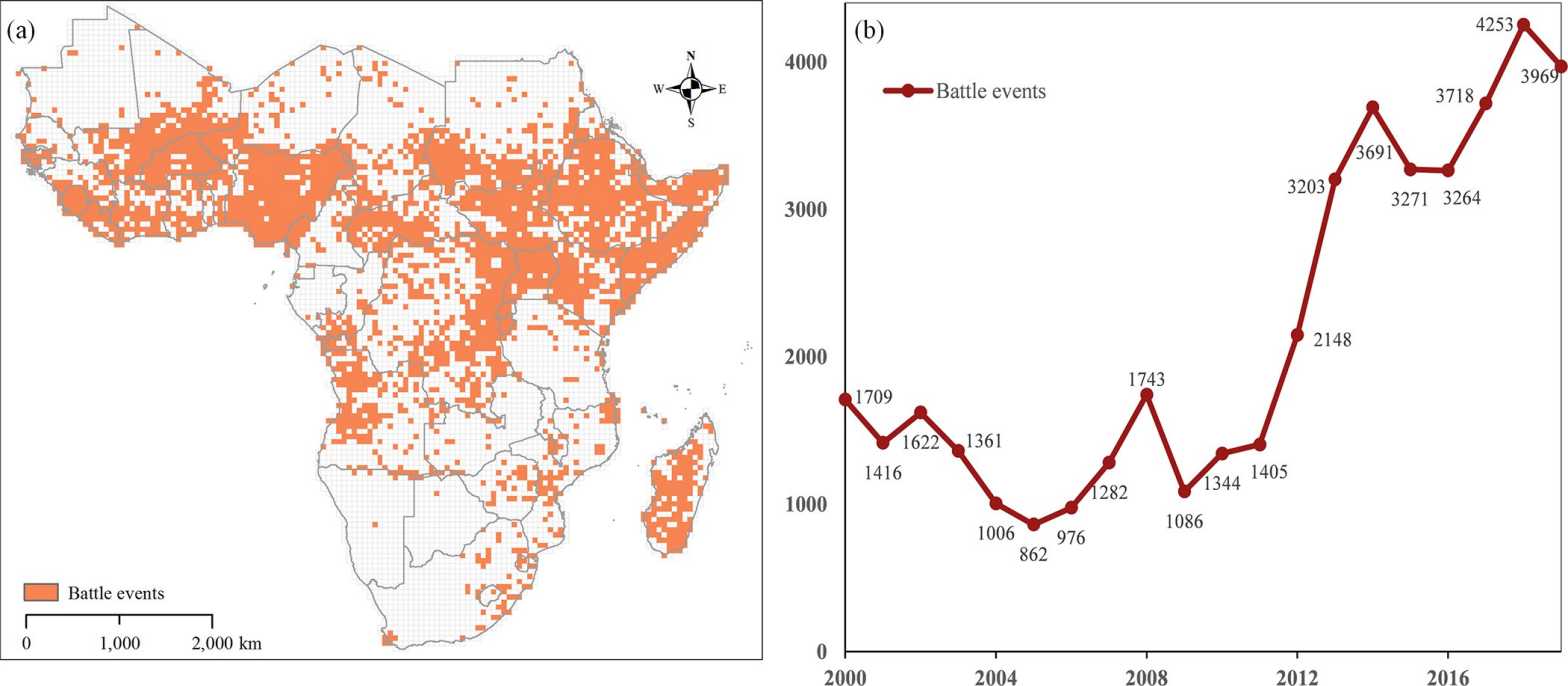
The spatial distribution (a) and annual number statistics of (b) battles events from 2000 to 2019.

In Fig 1(A), we map the spatiotemporal distribution of battle events from 2000–2019 in sub-Saharan Africa. The map clearly shows that during the last 20 years, the battle events tend to be localized, clustering in particular places such as West Africa, the African Great Lakes region, and the Horn of Africa. The annual number of battle events from 2000–2019 is shown in Fig 1B), which indicates that the number of battle events presents a U-shaped distribution from 2000–2008. Subsequently, the number increased greatly from 1086 in 2009 to 3691 in 2014. After 2015, the number of battle events began to fluctuate.

### 3.2. Accuracy evaluation

**Fig 2:**
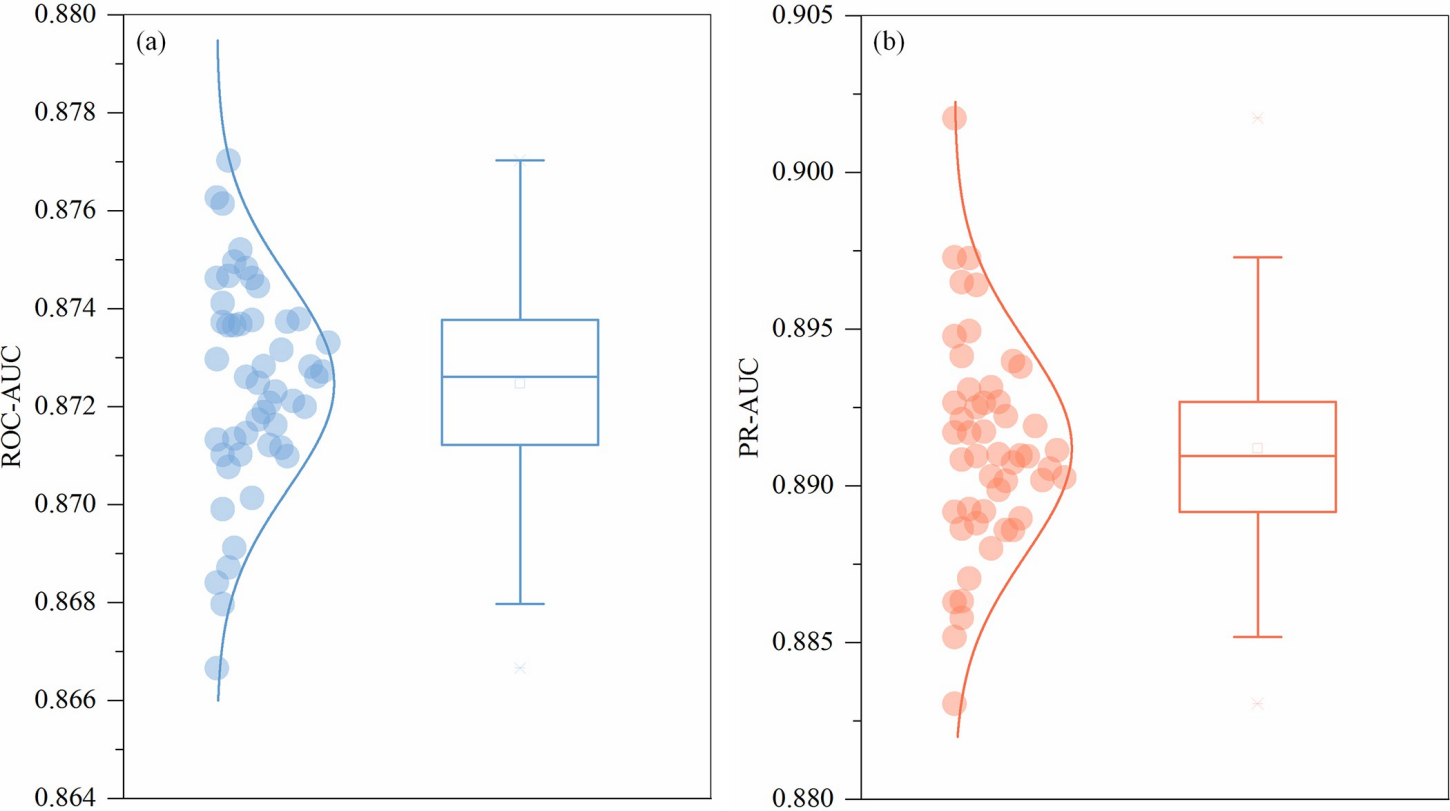
Validated performance of the boosted regression tree models by using the accuracy evaluation indices ROC-AUC (a) and PR-AUC (b).

Based on the datasets mentioned in the Data section, we establish a BRT model to measure the relative contribution of each covariate and simulate the incidence risk of battle events. Due to model randomness, we built 50 simulation processes for each BRT to evaluate variability across runs. Fig 2 describes the accurate evaluation of the BRT models trained on all samples. From Fig 2, we can see that the ROC-AUC values of the 50 models ranged from 0.867 to 0.877, and the average was 0.872 (Fig 2(A)). Similarly, the PR-AUC values ranged from 0.883 to 0.902, and the mean value was 0.891 (Fig 2(B)). In general, the values of these two accuracy evaluation indices for BRT models remain relatively high, which represents a good overall predictive performance. Moreover, the accuracy verification index value of the BRT model is slightly higher than that of the baseline model (ROC-AUC:0.844, PR-AUC:0.851), which again shows the superiority of BRT in conflict simulation.

### 3.3. The relative contributions of the driving factors

Table 2 shows the relative contribution (RC) of each driving factor after 50 ensemble BRT modelling analyses. The background context factors are the most important predictor variables in the ensemble models, with a mean relative contribution of 92.599%. Among them, the relative contributions of population density (RC 30.311% ± SE 1.005%), the liberal democracy index (RC 23.494% ± SE 0.612%), and urban accessibility (RC 14.217% ± SE 0.522%) are more than 10%. Others are, by decreasing order of their RC to the BRT ensemble, mean precipitation (RC 9.178% ± SE 0.562%), mean temperature (RC 8.262% ± SE 0.432%), normalized difference vegetation index (RC 4.353% ± SE 0.248%), nighttime lights (RC 2.12% ± SE 0.16%), exclusion (RC 0.512% ± SE 0.074%) and land cover (RC 0.152% ± SE 0.078%). By comparison, the climate change-related variables have relatively little effect on the spatiotemporal distribution of armed conflict risk, accounting for less than 8% of the total: temperature anomaly (RC 4.658% ± SE 0.237%) and precipitation anomaly (RC 2.743% ± SE 0.181%).

**Table 2:** The relative contribution of the related spatial predictor variables.

Variables	Relative contribution ± Standard Deviation, %
**Background contexts**	**92.599**
Population density	30.311±1.005
Liberal Democracy Index	23.494±0.612
Urban accessibility	14.217±0.522
Mean precipitation	9.178±0.562
Mean temperature	8.262±0.432
Normalized difference vegetation index	4.353±0.248
Nighttime lights	2.120±0.160
Exclusion	0.512±0.074
Land cover	0.152±0.078
**Climate change**	**7.401**
Temperature Anomaly	4.658±0.237
Precipitation Anomaly	2.743±0.181

### 3.4. Spatial distribution of armed conflict risk

**Fig 3:**
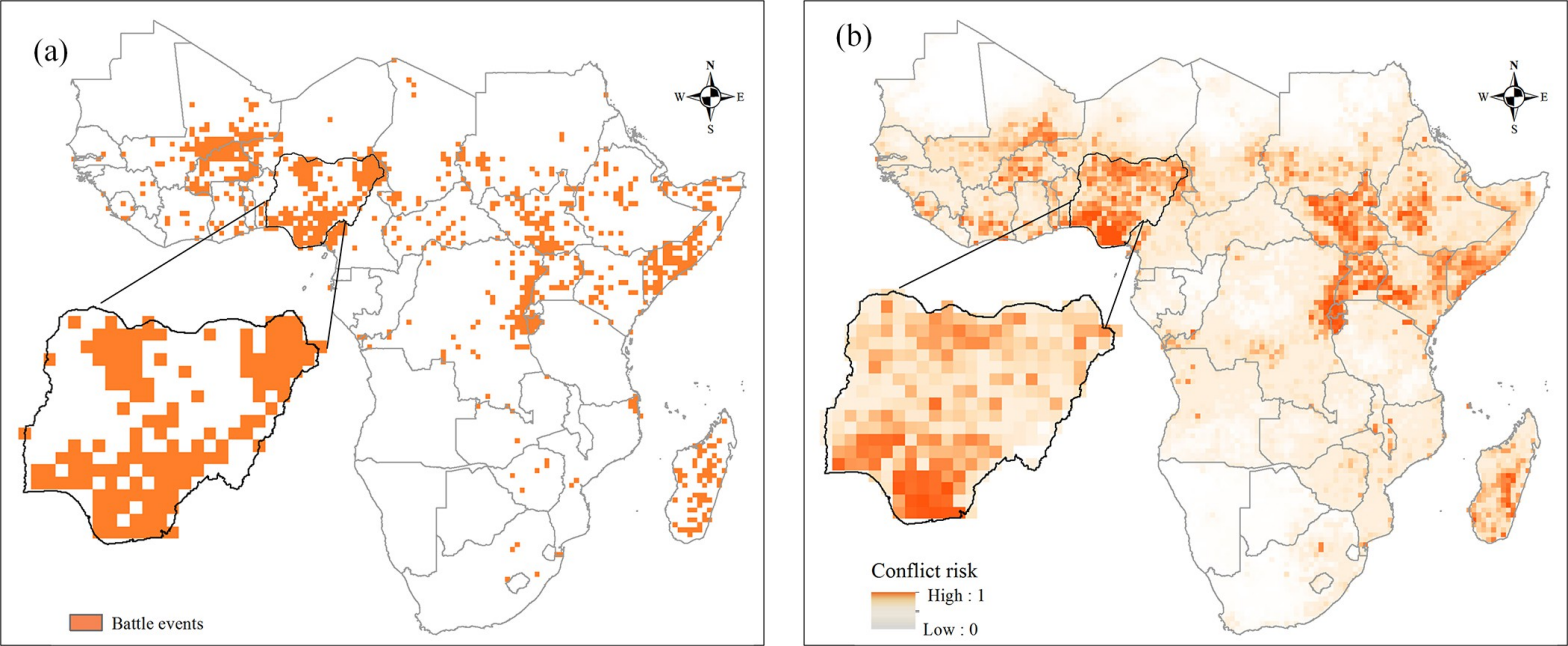
The spatial distribution of battle incidence in 2019 from the actual record (a) and simu-lated by the ensemble BRT Models (b).

We adopted ensemble BRT models to simulate the risk of incidence of battle from 2000 to 2019. Fig 3(B) depicts the simulated probability of battles in 2019 based on the 50 ensemble BRT models trained on all incidence samples at a 0.5° × 0.5° spatial resolution. The results show that the simulated higher-risk areas of incidence of battle are concentrated in the Horn of Africa, Uganda, South Sudan, the northeastern part of the Democratic Republic of the Congo, and Nigeria, which is generally consistent with the actual record of battle events (Fig 3(A)). Although the BRT models established by us can precisely simulate the highest risk of incidence of battle, after carefully comparing Fig 3(A) with Fig 3(B), we find that the predictive performance of the BRT model is relatively low for some scattered points. For example, there tends to be overstimulation of battles incidence in places such as Angola and Malawi. The risks of some zones, such as the northwest corner of Mozambique and the Central African Republic, are underssimulated.

## 4. Conclusion and discussion

Understanding the driving factors and establishing the simulation model of armed conflict have long been considered a core task for peace research [55]. This study used datasets of armed conflict, climate change, and background context for the period of 2000–2019. Using a machine learning method to establish a comprehensive simulation modelling approach for armed conflict in sub-Saharan Africa at a fine spatial scale of 0.5° serves as a reference for determining the relative importance of factors influencing the prediction of future armed conflict.

This study includes the possible driving factors of armed conflict from two aspects and estimates the relative contribution (RC) of each factor. The results show that the mean RC of climate change and the background contextual factors (representing politics, socioeconomic status, and the environment) are 7.4% and 92.6%, respectively. We note, the RC of climate change is in the lower range of, but in line with a group of experts’ judgments that 3–20% of conflict risk is affected by climate change [16, 55]. Though the RC values of some factors are slightly different compared to the impact scores calculated in the baseline model (S1 Table) and previous work, they consistently indicate how modest the contribution of climate change-related factors to the spatiotemporal distribution of armed conflict incidence is relative to the background context factors like political, economic, and physical geographic [21, 56, 57]. For example, Ge et al., [57] showed that stable background covariates (RC>96%) are the main factor influencing the distribution of armed conflicts, while climate change (RC<4%) had relatively little effect on the armed conflict simulated results. By contrast, the impact value of climate change on conflict risk estimated in this study is greater than the value estimated in the above global study, which revealed conflict in Sub-saharan Africa is more sensitive to climate change compared to other regions [58].

Researchers have been studying the formation of conflict occurrence for a long time and various statistical analysis and machine learning techniques have been used to forecast conflict [59–61]. These studies reveal the potential of data-driven analysis and machine learning for modeling and predicting conflict [41, 62], which laid a foundation for conflict research. Based on these previous studies, this study uses BRT models to simulate the incidence risk of armed conflict by assembling a large number of armed conflict events along with multiple variables that are generally accepted as important armed conflict risk factors [63, 64]. The ensemble BRT models obtain a good simulation accuracy with a mean ROC-AUC of 0.872 and PR-AUC of 0.891, which indicates that the BRT model established in this study can simulate the incidence of most armed conflict precisely. This result further indicates the potential of the machine learning model in armed conflict risk projections [57].

Although this study comprehensively considers the driving factors of armed conflict and establishes a BRT modelling approach to simulate the areas at risk of armed conflict, it is essential to point out several possible limitations of our analysis that should be improved in the future. First, this study only focused on the battle events of armed conflict; other types of conflict (i.e., protests and riots) are not considered, which should be further explored in future studies. In addition, more research is needed to explore the interactions of driving factors to better understand their indirect effect on armed conflict. Moreover, although the ensemble BRT models can better simulate conflict events with obvious spatial agglomeration characteristics, they have limited capacity to capture the characteristics of scattered points. With the improvement of data acquisition means and machine learning approaches, new data and insights can be added to improve the ability of conflict simulation and prediction.

## Supporting information

S1 Fig Cross-validation model-fitting example. An initial number of trees = 50, step size = 10. With a learning rate of 0.01 and a tree complexity of 4, the step procedure identified the optimal number of trees as 3500.
(TIF)

S1 Table The relative contribution of the related spatial predictor variables is estimated by random forest. (DOCX)
