# Supplementary figures and images for: Modeling analysis of armed conflict risk in sub-Saharan Africa, 2000–2019
Source: PLoS One. 2023 Oct 2;18(10):e0286404. doi: 10.1371/journal.pone.0286404 (PMC10545108; doi:10.1371/journal.pone.0286404)

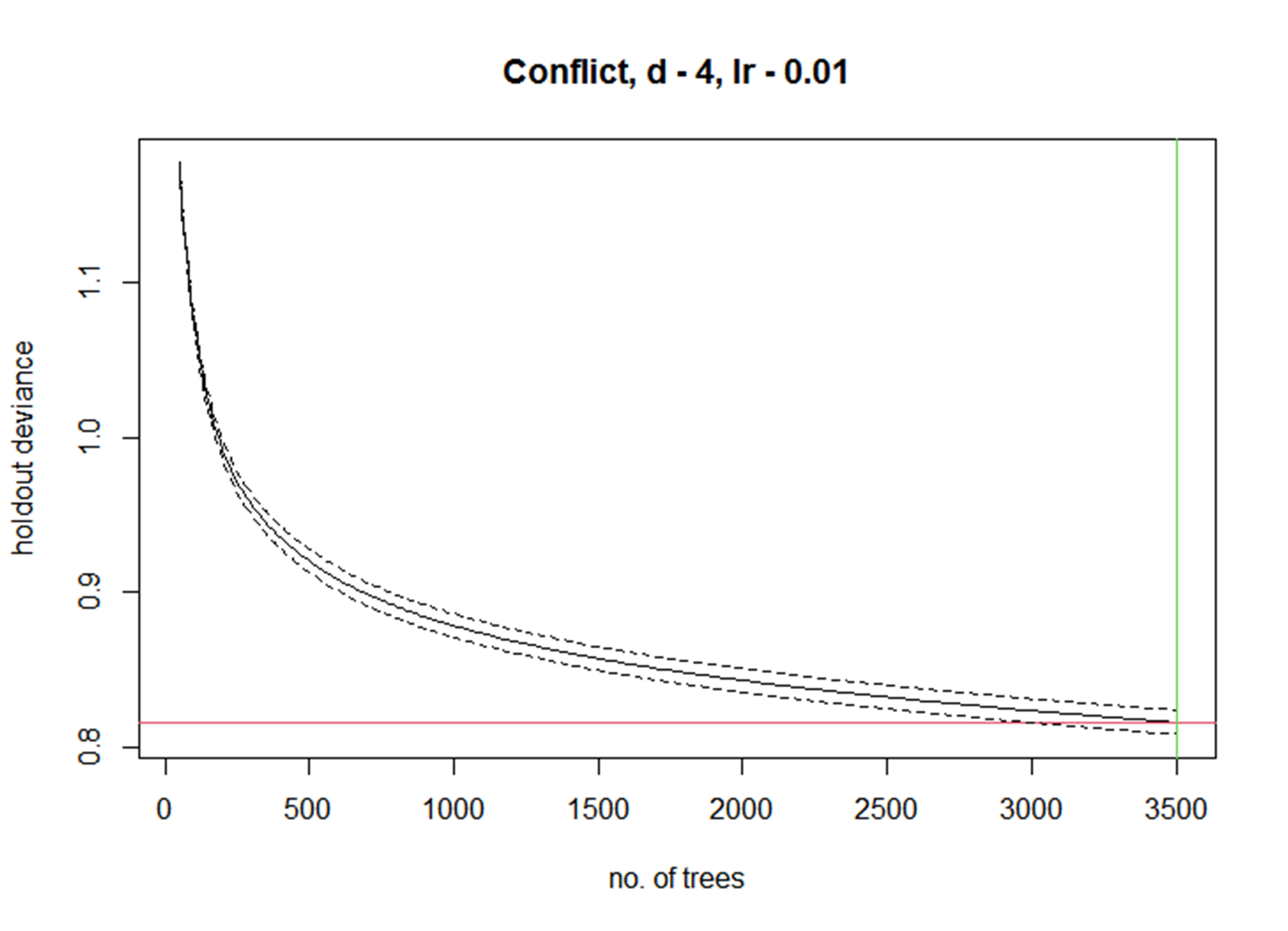

Supplement: S1 Fig — An initial number of trees = 50, step size = 10. With a learning rate of 0.01 and a tree complexity of 4, the step procedure identified the optimal number of trees as 3500. (TIF) [file pone.0286404.s001.tif]
