# Supplementary material for: Modeling analysis of armed conflict risk in sub-Saharan Africa, 2000–2019
Source: PLoS One. 2023 Oct 2;18(10):e0286404. doi: 10.1371/journal.pone.0286404 (PMC10545108; doi:10.1371/journal.pone.0286404)
Supplement: S1 Table — (DOCX) [file pone.0286404.s002.docx]

**Table S1.** The relative contribution of the related spatial predictor variables is estimated by random forest.

| Variables | Relative contribution, % |
| --- | --- |
| **Background contexts** | **90.201** |
| Population density | 33.347 |
| Liberal Democracy Index | 17.265 |
| Mean precipitation | 10.438 |
| Mean temperature | 10.184 |
| Urban accessibility | 10.039 |
| Normalized difference vegetation index | 4.464 |
| Nighttime lights | 2.046 |
| Exclusion | 1.305 |
| Land cover | 1.113 |
| **Climate change** | **9.07** |
| Temperature Anomaly | 5.88 |
| Precipitation Anomaly | 3.19 |
